# Modulatory Effect of Dietary Polyunsaturated Fatty Acids on Immunity, Represented by Phagocytic Activity

**DOI:** 10.3389/fvets.2020.569939

**Published:** 2020-09-22

**Authors:** Hanan Al-Khalaifah

**Affiliations:** Environment and Life Sciences Research Center, Kuwait Institute for Scientific Research, Kuwait City, Kuwait

**Keywords:** immune status, nutrition, phagocytosis, polyunsaturated fatty acids (PUFA), lipid

## Abstract

Lately, dietary polyunsaturated fatty acids (PUFAs) have shown substantial importance in human and animal nutrition, especially those of the *n*-3 group. Development and optimal functioning of the immune system are directed affected by diet. These dietary fatty acids have an important impact on the health and immune competence of various species including human beings. They are essential for the modulation of immune responses in health and disease. Fatty acid composition of immune cells can be modulated by the action of dietary fats and the outcomes in the composition can produce functional effects on reactivity and functioning of immune cells in a short period. There are several mechanisms involved in impacting dietary fatty acids on immune function; however, lipid mediator synthesis from PUFAs is of great importance in terms of inflammation. The objectives of this article are reviewing studies on the impact of PUFA in the diet on phagocytosis of chickens, murine, rats, ruminants, and humans. It also sheds light on the possible mechanism by which this immunomodulation occurs.

## Introduction

A diet rich in nutrients is of utmost importance for the proper functioning of a living organism including its immune system. Certain dietary constituents possess immune-regulatory properties such as vitamins and fatty acids (FAs). For numerous years, researchers have been studying the impact of dietary polyunsaturated fatty acids (PUFAs) on the immune status with an emphasis on omega-3 PUFAα-linolenic acid (ALA), eicosapentaenoic acid (EPA), and docosahexaenoic acid (DHA) (1). PUFAs have been widely used as supplementary ingredients in the feed rations of animals, as well as in humans to improve the health status and the immune function (2–8). PUFAs are broadly divided into two types: omega-3 (*n*-3) FAs, originated from ALA; and omega-6 (*n*-6), obtained from linolenic acid (LA) (4, 6, 9–21). Dietary imbalance in the ratios of *n*-6/*n*-3 (PUFAs) could adversely influence human and animal health. These FAs are known to alter immune cell functions as they change the makeup of cell membrane phospholipids to membrane lipid fluidity, signal transcription factors, and bioactive synthesis of lipid mediators. PUFAs along with maintaining cell structure and function also enhance immune functions, promote growth and maturity, and regulate lipid metabolism and related gene expression (22, 23).

The objectives of the present paper are reviewing studies on the effect of supplementary PUFA in the diet on phagocytosis of chickens, murine, rats, ruminants, and humans. It also sheds light on the possible mechanism by which this immunomodulation occurs.

The process of phagocytosis is essential to provide nutrients to unicellular and multicellular species where it takes place through specialized cells called phagocytes. These cells recognize and engulf elements of more than 0.5 μm through the plasma membrane by forming a phagosome. Microbial pathogenic agents and apoptotic cells can also be ingested by phagocytes. Hence, they not only eliminate microbes but also play a role in tissue homeostasis (24). This phenomenon was first observed in polymorphonuclear neutrophils and macrophages by Elie Metchnikoff who was named as the innate immunity father. Broadly, the cells involved in phagocytosis can be divided into two classes: specialized and non-specialized phagocytic cells. The former involves polymorphonuclear neutrophils, monocytes, monocyte-derived macrophages, and tissue-resident macrophages whereas the latter consists of epithelial cells, fibroblasts, and dendritic cells (DCs). These non-specialized cells are limited to specific targets and are slow in function. Besides playing a role in innate immunity, both specialized and non-specialized phagocytes are also are required for maintaining a strong physiological status, in rescue from damage, and for the development of the host animal (25).

Phagocytosis involves several receptors that can be categorized as non-opsonic and opsonic receptors. Non-opsonic receptors have to ability to recognize molecular groups on the cell membranes of the target cells. Examples of these receptors are lectin-like recognition molecules such as CD169 and CD33; C-type lectins such as Dectin-2, Mincle, or DNGR-1; scavenger receptors; and Dectin-1, which is a receptor for fungal beta-glucan. Toll-like receptors (TLRs) detect foreign bodies but are not functional as phagocytic receptors. They combine with some non-opsonic receptors to trigger ingestion. Opsonic receptors identify opsonins that recognize foreign particles and ingest them. The most relevant receptor under this type is Fc receptors (FcR) and the complement receptors (CR) (24).

## Brief Overview of Cell Subtypes of Phagocytosis

Phagocytosis of microorganisms is predominantly carried out by two elements of the innate immune system, namely, macrophages and neutrophils (26). They are the first to respond to any tissue injury or infection. Traditionally, neutrophils were considered to be terminally differentiated cells that had antimicrobial functions. However, years of research have shown neutrophils in a different light such as they have a range of cytokines and effector molecules. They are also involved in numerous effector functions and can regulate the innate and adaptive immune system (27). Neutrophils are also known to have an important role as a modulator of inflammation and cancer immunology. The phagocytic capacity of neutrophils is dependent on age and disease in humans; this has been well-observed by an increase in neutrophil phagocytic activity in patients with rheumatoid arthritis (26). Neutrophils detect danger signals through the action of pattern recognition receptors (PRRs) and pathogen- or danger-associated molecular pattern (PAMP and DAMP) receptors that travel across the vascular endothelium to reach the location of infection and then ingest and kill the infectious agents. Migration of neutrophils occurs by stages that begin when selectin and chemokine receptors are encaged and used for activation of integrin-dependent arrest and migration. On arrival, neutrophils engulf alien elements and eliminate them into the vacuoles that obtain enzymatic and oxidative properties through fusion with secretory granules that contain bactericidal enzymes and a superoxide-generating NADPH oxidase. Also, these cells produce superoxide and DNA bound to histones and granular proteins to kill the infectious agent. It was revealed that the level of cytosolic Ca^2+^ controls the functionality of the neutrophils. This is achieved by controlling selectin, chemokine, and integrin signaling networking that induces neutrophil binding, scattering, and migration. It also helps in the remodeling of the actin cytoskeleton and integrin reprocessing during the movement process. Ca^2+^ concentrations also control the exocytosis of neutrophil granules and the manufacturing of superoxide by NADPH oxidase enzyme present in the plasma membrane and phagosomes. The phagocytic ingestion phase largely relies on Ca^2+^ elevations in neutrophils based on the phagocytic receptors involved (28).

Monocytes are leukocytes that differentiate into macrophages and myeloid lineage dendritic cells. Monocytes along with macrophages and dendritic cells perform three main functions which are phagocytosis, antigen presentation, and cytokine production. Monocytes circulate in the blood for about 1–3 days after which they move to tissues in the entire body and differentiate into macrophages and dendritic cells. They make up 4–10% of leukocytes in the blood (29).

Besides acting on microbial agents, macrophages are also responsible for maintaining homeostasis of the body by the removal of internal waste materials and tissue repair. Macrophages use antibody-dependent cellular phagocytosis to eliminate bacterial and fungal pathogens and also virally infected host cells (30). Those macrophages that are activated by the attack of pathogens and can eliminate them are known as M1 macrophages, and those that cause chronic allergy and inflammatory disorders, fat metabolic pathways, wound curing, and cancerogenic attack are known as M2 macrophages. It was reported that theM1 macrophages are stimulated by PAMPS, DAMPs, and inflammatory cytokines like TNF-α and IFN-γ, whereas M2 macrophages are stimulated by anti-inflammatory cytokines like IL-10, IL-4, and IL-13 (31). Macrophages like neutrophils kill pathogens by the production of enzymes and increasing the acidity in the phagosomes. It was observed that macrophages produce less ROS and quicker acid formation than neutrophils due to the quick delivery of V-ATPase. Also, macrophages can present antigens to other lymphocytes to stimulate the specific immune response (28). Monocyte-derived macrophages express a range of phagocytic receptors like the FcγRs and CRs for opsonized targets, scavenger receptors, and C-type lectins (e.g., mannose receptor and dectins) for fungal cells. Tissue macrophages have adapted to their surroundings where they exist according to their specific functions. Earlier, it was thought that macrophage cells in the different tissues undergo differentiation of hematopoietic stem cell. However, it was later discovered that embryo yolk sac macrophages are capable of producing macrophages in the liver, skin, and the central nervous system. These AMs can self-renew without the need for monocyte-derived macrophages, even after the reduction of AMs during influenza infection (25).

Dendritic cells (DCs) are antigen-presenting cells (APCs) obtained from hematopoietic stem cells from bone marrow. The ability of DCs to present antigens is to some extent dependent on phagosome maturation. DC phagosomes perform controlled proteolysis which is required for the production of peptides needed for adhering to the major histocompatibility complex molecules (MHC). The MHC has a critical role in presenting the antigen to the lymphocytes and in stimulating the specific immune response. Antigens are engulfed and destroyed into the phagosome. Sometimes, these antigens reach cytosol and are then transported into the endoplasmic reticulum where they link with MHC class I molecules; this is known as cross-presentation. Before activation, DCs express some receptors like FcγRs and scavenger receptors, plus TLRs (25). DCs use the ingested material from particles that were ingested by them and signal received to present antigens to naïve T cells and also help initiate T-helper, cytotoxic, or immunosuppressive responses. DCs have a short lifespan and are changed when the myeloid hematopoietic precursors are differentiated in the blood. Like neutrophils and macrophages, DCs also respond to PAMPs and DAMPs through PRRs which differentiate precursors into mature and immature DCs (28).

## Classification of Polyunsaturated Fatty Acids (PUFAS) and Their Dietary Sources

Fatty acids are the building blocks as they make up the structural components of cells, tissues, and organs. Unsaturated fatty acids (UFAs) are classified into monounsaturated fatty acids (MUFAs) and polyunsaturated fatty acids (PUFAs), the two important groups of PUFAs being omega-3 and omega-6 FAs. As mentioned previously, ALA, EPA, and DHA are the well-known members of the omega-3 family. ALA is the only omega-3 that is obtained through the diet as it cannot be synthesized by the human body. It is present in vegetable oils such as flaxseed, canola, soybean, and hemp oil, nuts, such as walnuts, and also in chia seeds, dairy products, eggs, and algae. These omega-3 fatty acids can also be found in the meat of free-range animals. ALA behaves as a precursor compound for the synthesis of other omega-3 fatty acids. Even though EPA and DHA can be derived through food, ALA can be converted to EPA and DHA in the body and can thus control their physiological activity. EPA and DHA are most commonly obtained in fish and seafood such as in fatty fish oils, squid and krill, egg oil, and seaweed, and therefore are also referred to as marine “omega-3s.” Omega-6 FAs are mainly a derivative of linoleic acid (LA). Just as in the case of ALA, LA too cannot be synthesized by the human body and has to be obtained through the diet. Ingestion of LA is followed by conversion into arachidonic acid (AA). Important plant dietary sources of omega-6 are corn, soybean, sunflower oil, coconut and coconut oil, almond, pine nuts, and hazelnuts. Some animal dietary sources are lard, turkey fat, and butter. PUFAs are essential for the development and smooth functioning of the brain and heart along with all tissues and organs. Deficiency of omega-6 LA results in poor growth, fatty liver, skin lesions, and reproductive failure. Some studies in rodents have shown the significance of n-3 PUFA deficiency in learning, memory, cognition, and behavior (32).

## Mechanism of Immunomodulation

Supplementary dietary FAs may influence the immune status via several mechanisms such as inhibition of arachidonic acid (AA) metabolic process, production of anti-inflammatory mediators, modification of intracellular lipids, and activation of nuclear receptors. Earlier research in animals and humans have shown that the type and quantity of fatty acid are linked to the effect on immune response (33). PUFAs have been widely used as supplementary ingredients in the feed rations of animals, as well as in human feed to improve the health status and the immune function (4, 5, 34–36). Lipids are also the main constituents for the central nervous system (CNS) with AA (20:4n-6) and docosahexaenoic acid (DHA, 22:6n-3) being the supreme significant. They are mostly present as phospholipids and help in the formation of brain cell membranes. One of the most preferred mechanisms to emphasize the immunomodulatory properties of n-3 long-chain PUFAs is the production of bioactive fat derivatives or oxylipins. First, those eicosanoids are responsible for regulating inflammation like prostaglandins, leukotrienes, thromboxane, and those involved in controlling the inflammatory reaction and are called Specialized pro-resolving Mediators (SPMs) (i.e., resolvins, protectins, maresins). These SPMs can induce anti-inflammation and pro-resolution characteristics without suppressing the immune status and restore homeostasis. They are involved in the downregulation of cytokines that are involved in pro-inflammation and upregulation of those involved in anti-inflammation. These SPMs also induce phagocytic cells against debris and lifeless cellular components and decrease levels of oxylipins that induce pro-inflammation (37–39). The enzymes required for synthesis include phospholipases A2 (PLA2s), in addition to cyclooxygenases (COX), lipoxygenases (LOX), and cytochrome P450 (CYP450) (37). These are also responsible for switching DHA and EPA into biologically active mediators.

The human serum consists of 30.7 and 25.9% of known SPMs of DHA and EPA derivatives, respectively. COX-2 is expressed in the hippocampus after an inflammatory stimulus like lipopolysaccharide (LPS). Inhibition by COX-2 slows down the occurrence of critical inflammatory response. CYP450 induces the production of epoxides that are related with anti-inflammation. From DHA, various SPMs have been synthesized, namely, monohydroxy DHA (17-HDHA) by acetylated COX-2, CYP450, and 15-LOX and resolvin D1 (RvD1) through the synthesis of 17-HDHA by 5-LOX. When RvD1 was studied in mouse models for cerebral ischemia, its levels were changed by a DHA intravenous supplementation. The receptor of RvD1 is lipoxin A4 receptor/formyl peptide receptor 2 (ALX/FPR2) in rodents and G protein coupling receptor 32 (GPR32) in humans. EPA is altered by acetylated COX-2 or CYP450 into 18R-HEPE, which is then broken down into resolvins. The resolvin RvE1 brought about a reduction in LPS-provoked cytokines that stimulate pro-inflammation (TNF-α, IL-6, IL-1β) and gene expression in microglial cells by interfering with the NFκB signaling route. The receptors of RvE1 are a G protein coupling receptor, ChemR23, or chemokine-like receptor 1 (CMKLR1) and a leukotriene B4 receptor (BLT1). Omega-6 linoleic acid (LA) and AA are both precursors of EPA and DHA and are solely supplied by food sources. Deficiency of omega-3 and excess of omega-6 PUFAs are linked to various metabolic diseases. Intake of ARA is linked with obesity, inflammatory disease, and associated metabolic syndrome. Omega-6 oxylipin-oxygenated derivatives of PUFAs support inflammatory responses, assist in energy storage, and inhibit energy expenditure. The n-6/n-3 PUFA proportion is more critical than the entire PUFA intake as this ratio determines the level of synthesized n-6–derived oxylipins (37).

The n-3 PUFAs, EPA, and DHA are known to be most beneficial for mammalian species in terms of health and nutrition and for their anti-inflammatory properties. The n-3 PUFAs change the immune response through numerous pathways such as swapping AA during eicosanoid signaling cascade; this decreases the production of eicosanoids that induce inflammation such as PGE2, TXB2, and LTB4. They also interfere with cytokine gene expression. As monocytes are the main effector cells of the immune system, long-chain n-3 PUFA influence monocyte/macrophage defensive functions; both EPA and DHA can enhance phagocytic activity and decline chemotaxis of monocytes in humans (40).

Studies in literature show that the effects of PUFA on phagocytic activity are inconsistent and debatable. Studies report that PUFA incorporation diminishes, enhances, or has no effect on phagocytic activity. Rodent, human, and chicken studies are discussed separately below.

## PUFA'S Influence on Immune Cells

Macrophages can recognize specific PAMPs due to the presence of TLRs. Once the pathogen is recognized, the start of its elimination is activated by phagocytosis and reactive oxygen species (ROS). They also manufacture and discharge various cytokines and chemokines for the destruction of the pathogen. These activated macrophages, also known as M1 macrophages, release TNF-α and IL-1β which further aids in the elimination of the pathogen. Curing macrophages with EPA or DHA triggers many alternations in the gene expression in THP-1–derived, LPS-activated macrophages. N-3 PUFAs reduce M1 polarization of macrophages; however, ALA, EPA, and DHA increase phagocytosis by macrophages. This has been depicted in the phagocytic capacity as seen in swallowing up of the target live and dead cells. Researchers have reported that these events could be due to alternations in cell membrane confirmation due to FAs (1).

Neutrophils are found in abundance in humans particularly in circulation, blood, bone marrow, spleen and liver, and certain tissues. The n-3 PUFA combines with phospholipids in the membranes of neutrophils at the cost of n-3 PUFAs: LA and AA. In doing so, these can undergo specific metabolic pathways by neutrophils into prostaglandins, leukotrienes, thromboxanes, maresins, protectins, and resolvins. The n-3 PUFAs are known to boost neutrophil phagocytic activity in mice. The addition of DHA *in vitro* to separated peritoneal neutrophils showed a 35% rise in phagocytic and fungicidal capacity. In goats, polymorphonuclear leukocytes when incubated along with EPA or DHA enhanced its ability to engulf *E. coli*. This is partially true for humans as well. When 10 participants were supplemented with fish oil (FO) comprising 26% EPA and 54% DHA every day for 60 days, the neutrophil phagocytosis was observed to increase by 62%. Another study where the volunteers were supplemented with corn oil or EPA showed no effect on phagocytic capacity. Dendritic cells have been well-studied as APCs in the functioning of the specific immune system. In human and mouse dendritic cells, there was a downregulation of the MHC II due to the effects of EPA and DHA. Also, the T-cell activation by n-3 PUFA-cured dendritic cells was severely affected (1).

Mast cells are normally associated with response to allergies. These cells attach to IgE produced by B cells via their high-affinity IgE receptor. In many human and animal models, it has been observed that omega-3 FAs lowers IgE-mediated activation of mast cells. The diminishing effect of these FAs is used to lower the long-term effects of allergies or atopic dermatitis (1).

Interaction with TLRs leads to the stimulation of signaling pathways that activate the synthesis of cytokines responsible for anti-inflammation and simultaneously stopping the synthesis of cytokines that are responsible for pro-inflammation. Interaction of FAs with TLRs, especially TLR2 and TLR4, on lymphocytes showed that saturated FAs increased COX-2 expression and phosphorylation of ERK (p-ERK). Omega-3 FAs and DHA on the other hand caused suppression of COX-2 and p-ERK. Monocytes like THP1 and macrophages like RAW 264.7 showed an elevated response to FAs in serum-deprived conditions determined by ROS in the microenvironment whereas EPA and DHA decreased the response. High-fat diets rich in these FAs have been known to increase endotoxin (LPS) and TLR4 activation. Saturated FAs also cause the activation of other pro-inflammatory pathways concerning intracellular macromolecular complex Nod-like receptor pyrin domain-containing protein (NLRP)-3 inflammasome, which produces cytokines such as IL-1β and IL-18. Another type of membrane receptor is PPAR-gamma (PPAR-γ). It is handled in membrane lipid rafts and has a critical action in inflammation in an NF-κB–dependent way. Cell surface receptor-like PPAR-γ triggers the cascade for the synthesis of lipid moderator like prostaglandins and cytokines (like IL-2). Suppression of NF-κB–mediated pro-inflammatory responses by EPA and DHA is unconnected to PPAR-γ (41).

## Studies in Chickens

Poultry possesses the ability to accumulate PUFAs in their tissues when fed diets rich in sources of PUFA. The results showed that fatty acid incorporation in the tissues and blood plasma indicated the fatty acid composition of the dietary rations (34, 42–46).

Studies in the literature regarding the immunomodulation effect of PUFA on phagocytosis are quite limited. Supplementation of poultry diets with FO, which is a rich source of PUFAs such as EPA and DHA, has been found to have nutritional and immunomodulatory effects. In birds, the immunomodulatory effect of PUFA occurs as a result of intercellular communications and signals that affect the reactivity of leukocytes as a result of antigenic motivation. This is about downregulation or upregulation of various cytokines that are claimed to affect the avian immune system such as IL-1β, IFNγ, MGF, IL-1, IL-4, and IL-2. Phagocytosis of avian heterophils and mononuclear cells can be detected using phagocytosis assay and certain bacteria such as *E. coli*, SRBC, and yeast cells. Opsonization of these bacteria by complements and/or antibodies is usually conducted to stimulate the phagocytic activity (13, 47–50). Poultry feed ration fortified with FO may have the ability to change the avian immune system and thus influence the bird's ability to fight infectious disease agents. For example, the percentage of monocytes engaged in phagocytosis was decreased in broilers fed on 60 g/kg (9.87% EPA and 12.92% DHA of the total FO) of FO in the feed ration, relative to the standard group with no FO supplementation (4, 51).

In hens fed FO and linseed oil–supplemented diets, antibody titers were found to be greater in comparison with those fed corn oil. It is also known that PUFA can inhibit the production of cytokines. It was seen that the addition of FO enriched with n-3 PUFA in broiler diets showed an inflammatory inhibition and lowered the plasma tumor necrosis factor (TNF) and interleukin-1 (IL-1) from peripheral macrophages (4). Shawky et al. (52) assessed the effects of linseed oil and fennel oil on the immunity of 90 Cobb broiler chickens. The dietary treatments included a basal diet (control), a basal diet supplemented with 0.2 ml linseed oil, and a basal diet supplemented with 0.3 ml fennel oil. Significant results were observed, and there was an increase in WBCs, heterophil%, phagocytic activity%, phagocytic index, total protein, globulins, IgM, IgG, A/G ratio, T3, and T4 of broiler chicks compared with the control group for birds fed linseed oil and fennel oil supplemented diets, thus showing that the inclusion of both these oils could improve the immunity of the birds. Another study made use of FO, coconut oil, canola oil, and a mixture of the three oils in 250 unsexed 1-day-old broiler Cobb chicks. The observations showed improvement in feed conversion ratio and reduction in abdominal fat. High-density lipoprotein cholesterol increased whereas low-density lipoprotein cholesterol and malondialdehyde decreased. Immunoglobulin IgG and IgM also increased due to coconut, canola, and a mixture of the oils and IgG increased due to FO. The immunomodulatory effect of dietary PUFA on broiler chickens has been extensively studied although its effect on phagocytosis is very limited with inconsistent results. Some studies have shown that FO and linseed oil lowers the production of pro-inflammatory leukotrienes and can enhance certain immune responses in growing chickens. The α2-globulin and γ-globulin also showed an increase due to FO and canola oil. Improvement was also noticed for overall antioxidant status (53).

## Studies in Murines and Rats

Murine studies revealed that dietary n-3 PUFA decreased, increased, or have no effect on engulfing the ability of phagocytic cells. Schroit and Gallily (54) cultured peritoneal MØ with labeled *Shigella flexneri*, palmitoleic, oleic, elaidic, linoleic, and α-linolenic acids, and AA. The authors showed that the phagocytic activity was increased from 184 ± 6 to 211 ± 6 for the control and the α-linolenic cultures, respectively; numbers express bacteria/macrophage/hour. The authors also showed that phagocytosis was increased in a manner which appeared to be correlated with the degree of unsaturation of the FA supplement. The degrees of unsaturation were 1.088 and 1.407 in the control and the α-linolenic supplemented cultures, respectively. They suggested that fluidity of the plasma membrane would be dependent essentially on the fatty acid and cholesterol composition of the lipid bilayer matrix, and possibly on secondary interactions of the lipid with protein elements. Increased fluidity was related to the increased phagocytic activity of the cells. In the study of Calder et al. (55), murine phagocytes were stimulated with zymosan and cultured with different types of FAs, complexed with bovine serum albumin. Results of this study revealed that all FAs, including n-3 PUFA, were incorporated into plasma membranes of cells and that macrophages exhibited 25–55% enhancement in their phagocytic capacity in the EPA and DHA cultures. Conversely, results showed that macrophages enriched with the SFAs, myristate or palmitate, showed decreases in their phagocytic capacity of 28 and 21%, respectively. The authors also related changes in phagocytosis activity to changes in membrane structure, in particular changes in the fluidity of the membrane markedly influenced by the lipid composition.

In a study by Stewart-Phillips et al. (56), mice were fed a normal or high-fat diet and their macrophages were stimulated with low-density lipoprotein. There was only a slight inhibition of phagocytosis by the high-fat diet (by only 15%, not significant). Benquet et al. (57) fed mice on 10 g/100 g linseed oil containing over 50% of 18:3 (*n*-3) α-linoleic acid, beef tallow containing mostly 18:1 (*n-*9) saturated fat, safflower oil, an 18:2 (*n*-6) PUFA, and FO, containing longer chain (*n*-3) PUFA (for 8 weeks). The peritoneal MØ of the exercised mice were stimulated with fluorescent microspheres and showed decreased phagocytosis in exercised mice fed linseed oil, but there was no effect of FO. In the study of Eicher and McVey (58), the authors fed mice on a 3 or 20% corn diet or FO diet (17%+3% corn oil) (for 4 weeks). Then 3.1 × 10^8^ of *S. typhimurium* was given orally. The authors showed that FO decreased Kupffer cell phagocytosis before infection, relative to the control diet (~55% vs. ~62%). In the same study, FO increased splenocyte phagocytosis at day 14 of infection, relative to the control diet (~75% vs. ~70%). Kew et al. (59) fed mice on nine diets containing 178 g lipid/kg and differing in the kind of n-3 PUFA and position of these in supplementary triacylglycerol. The standard diet had 4.4 g α-linolenic acid/100 g total FAs. In the other dietary feed rations, EPA or DHA replaced a proportion (50 or 100%) of the α-linolenic acid, and was in the sn-2 or the sn-1(3) position of triacylglycerol. The authors showed that EPA decreased monocyte phagocytosis when it was in the sn-1(3) position of dietary TAG, but not the sn-2 position, relative to the control (25 vs. 13.4%). EPA increased MFI (mean fluorescence intensity) for monocytes in the sn-2 position, relative to the control diet (360 vs. 325). The proliferation index was decreased for mice monocytes on feeding on 4.4 g/100 g EPA sn-1(3) diet vs. 4.4 g/100 g EPA sn-2 dietary feed ration (4,952 vs. 6,751). EPA also decreased neutrophil phagocytosis when it was in the sn-1(3) position (57.9 vs. 44.1%). Also, MFI was decreased for neutrophils from mice provided with EPA in the sn-1(3) position, relative to the control (252 vs. 182). Moreover, 4.4 EPA sn-1(3) diet decreased the index of phagocytosis by ~60% for neutrophils, relative to those fed on the control standard diet or 4.4 EPA sn-2 diets. There was no effect of DHA. The authors suggested that the sn-1(3) orientation of EPA increased the fluidity of the phagocyte cell membrane. The lack of effect of DHA in this study was explained by the authors based on DHA conformation because it functions contrary to the membrane-fluidizing impact due to its chain length and a high degree of unsaturation, whereas EPA increases membrane fluidity (60).

In a study by Hubbard et al. (61), mice were fed on 10% of either FO or safflower oil for 4 weeks. Results showed no effect of diet on peritoneal MØ stimulated with zymosan and sheep RBCs. Similarly, there was no influence of 68 mg/kg n-3 PUFA and 1,030 mg/kg vitamin E on blood phagocytes stimulated with labeled *E. coli* on adult BALB/c mice (62).

One *in vivo* study showed increased phagocytic activity after FO feeding (63). The authors fed mice on 9% by weight FO fortified with 1% corn oil (CO) containing linoleic acid (18:2n-6), 10% CO, or 10% borage oil (BO) containing 18:2n-6 and gamma-linolenic acid (18:3n-6). Zymosan was injected into the peritoneal cavity 1 h before sacrifice. The results showed that FO increased the proportion of MØ involved in phagocytosis: 62.2 vs. 32.6% BO- and 35.0% CO-fed group. FO also increased the mean number of particles ingested per MØ vs. BO, and CO (4.31, 3.51, and 3.57, respectively).

A more recent study sheds light on the immunomodulatory effect of DHA on immunosuppressive BALB/c mice model. The weights for spleen and thymus increased considerably by DHA (44.0 and 88.0 mg kg^−1^) supplementation in prevention or cure sets. In macrophages, there was enhanced propagation and phagocytosis in the prevention and cure groups due to DHA. Also, DHA activated macrophages by G protein–coupled cell membrane receptor GPR120–Mitogen-Activated Protein Kinases (MAPKs)–nuclear factor κB (NF-κB) p65 metabolism *in vivo*. In the spleen, DHA activated the proliferation of spleen cells and NK cells *in vivo*. The data for real-time PCR (qRT-PCR) revealed the production of cytokines IL-1β, IL-2, TNF-α, and IFN-γ in the spleen of immunosuppressive mice due to the presence of DHA. These findings are indicative of the immunomodulatory activity of DHA on immunosuppressive mice model in prevention or cure groups (64). Liu and Leung (65) studied the effects of jacaric acid, a conjugated linolenic acid (CLNA) isomer obtained from jacaranda seed oil on murine peritoneal macrophages. Their findings revealed that jacaric acid showed no significant cytotoxicity on murine peritoneal macrophages but increased cytostatic activity of T cells. Analyses by flow cytometry showed that jacaric acid enhanced the endocytic activity of macrophages. Also, there was an upregulation of pro-inflammatory cytokines such as interferon-γ, interleukin-1β, and tumor necrosis factor-α, thus showing that jacaric acid could exert an immunomodulatory effect on murine peritoneal macrophage cells *in vitro*.

Sepsis is one of the major reasons for passing away in intensive care units with passing away percentage of 30–70%. Gram-positive bacteria like *Staphylococcus aureus* play a major role in this infection. Antibiotics are used to counter this problem which has led to resistance by these bacteria. The activation of neutrophils is triggered as a defense mechanism against such bacterial infections. Studies have revealed that mice when fed high-fat dietary rations (HFDs) rich in PUFAs (HFD-P) have a higher frequency of neutrophils than mice fed HFDs rich in saturated fatty acids (HFD-S). N-3 PUFA metabolites called resolvins can enhance neutrophil capacity to phagocytose *E. coli*. The existence of mice was improved by diet with resolving D2 (RvD2). It was further investigated to check if HFD rich in omega-3 or mega-6 FAs is more effective at enhancing survival and lowering bacterial existence in mice with septic *S. aureus*–induced infection. The results showed that HFD-ω3–fed mice had higher survival rates than those fed HFD-ω6 or HFD-s, and there was a reduction in bacterial loads as well. In addition, HFD-S–fed mice also have a reduced frequency of phagocytosing neutrophils in their circulation. These findings showed that mice fed n-3 PUFAs have better ability to survive sepsis which is very likely due to an increase in neutrophils and precursor cells in the bone marrow and a restored phagocytic ability (66).

Numerous studies show the beneficial aspects of n-3 PUFAs in an inflammatory response in animals for acute and chronic inflammatory reactions, especially in the brain. Some studies in rodent models have shown that the resolvins, RvD1, and RvE1 show anti-inflammation in the CNS. RvD1 lowers the stimulation of NFκB and the production of elements involved in pro-inflammation such as IL-1β, IL-6, TNF-α, and iNOS in rats with hemorrhagic shock or streptozotocin-stimulated diabetic retinopathy. RvD1 reduces neuroinflammation by the ALX-FPR2 receptor through miRNA in a neonatal hypoxia–ischemia rat model. It also stimulates the accumulation of macrophages and microglia in the direction of M2 phagocytes. Persistent and early RvD1 supplementation in a rat modeling for Parkinson's disease can stop central and peripheral inflammatory responses. RvE1 helps reduce pro-inflammatory cytokines IL-1β and IL-6 in the prefrontal cortex and also in a murine model of Alzheimer's disease. It further regulates the stimulation of microglia by enhancing ramified microglia after traumatic brain damage. High concentrations of RvD1 in the brain in Fat-1 mice are associated with fewer cognitive defects, a decrease in microglial activation, and pro-inflammation due to high n-3 PUFAs in the brain during brain ischemia (37).

Data from rat studies were reviewed to study the influence of n-3 PUFA on phagocytic activity *ex vivo* and *in vitro*. There was one *in vitro* study which showed no effect of unsaturated FAs on phagocytosis of peritoneal macrophages *in vitro* (67). Overall, *ex vivo* dietary rat studies showed that n-3 PUFAs either increase or have no effect on phagocytosis. Four studies showed increased phagocytosis (68–71) and four studies showed no effect of PUFA on phagocytosis (72–75). One study showed that n-3 PUFA decreased phagocytosis in rats (76).

Cukier et al. (68) carried out a study to analyze the effect of total parenteral nutrition (TPN) on macrophage phagocytosis on Wistar rats. They were administered six varied types of oral isocaloric (1.16 kcal/ml), isonitrogenous (1.5 g/ml), and isolipidic (30% non-protein calories) TPN regimen diets. The rats were killed 96 h after TPN or saline infusion. The results showed that no significance was observed for liver, spleen, or lung weights. The non-lipid TPN inhibited macrophage phagocytosis of the spleen and lungs. The lipid TPN supplemented with fish oil emulsion showed a rise in total liver and lung macrophages. Thus, indicating that TPN supplemented with fish oil enhanced phagocytosis in rats. In the study of Bonatto et al. (69), FO increased phagocytosis by about 4-fold in the non–tumor-bearing rats, but there was no effect in the tumor-bearing rats. Female Wistar rats were supplemented with 1 g per kg body weight of either coconut oil or FO. This experimental treatment was repeated for two consecutive generations. At 90 days of age, male offspring (50%) from the F2 generation were injected with 2 × 10^7^ Walker 256 tumor cells in the subcutaneous layer. The results showed that FO ↑ phagocytosis by 30% in rats fed on a diet containing 1 g/kg FO vs. the control and coconut oil groups in the non–tumor-bearing rats. No influence was observed in the tumor-bearing rats (70). De Nardi et al. (71) administered jugular vein injections (for 5 days) with a mixture of 30% medium-chain triglycerides, 30% soybean, 25% olive, and 15% FO (emulsion 1); a physical combination of 50% medium-chain triglycerides and 50% soybean oil (emulsion 2); and 80% of the physical mixture supplemented with 20% FO (emulsion 3) or saline solution on 40 adult male Lewis rats. It was observed that 15% FO increased phagocytosis relative to the saline from ~23 to ~39 and from ~60 to ~100 in liver and lung, respectively. No effect was observed in the spleen. Moreover, 20% FO increased phagocytosis relative to the control group, non-surgical, from ~25 to ~49, ~59 to ~ 115, and ~160 to ~200 in the liver, lung, and spleen, respectively.

In the study of Magrum and Johnston (72), the authors fed rats on 10% corn oil or 10% linseed oil for 6 weeks and showed no effect of diet on phagocytosis of peritoneal MØ stimulated with yeast and carbon particles. Engels et al. (73) also showed no effect of FO on phagocytosis of peritoneal macrophages stimulated with antibody-coated erythrocytes in rats fed on FO for 8 weeks. Miyasaka et al. (74) fed rats on 0.4% of phosphate-buffered saline solution, cocoa butter, soybean oil, and FO and showed no effect of diet on phagocytosis of peritoneal MØ and mesenteric lymph node stimulated with opsonized zymosan. In the study by Palombo et al. (75), EPA administration did not affect phagocytosis of alveolar macrophages to zymosan. In the study of Bulbul et al. (76), DHA was melted in 1 ml of corn oil at a concentration of 36 mg/kg/day and the rats were orally administered for 4 weeks. Standard rats were given 1 ml/day corn oil as a standard. It was observed that DHA decreased the mean number of phagocytized particles per macrophage vs. corn oil, 6.14 ± 1.20, and 4.39 ± 0.44 particles/MØ for control (corn oil) and DHA, respectively.

N-3 PUFAs have previously shown to be effective in reducing tumor growth, particularly EPA and DHA derived from FO. These FAs present in the diet can alter the functions of the tumor and immune cells. Although the effects of ALA are controversial, 70-day-old non–tumor-bearing and tumor-bearing Wistar rats were supplemented with 1 g/kg body weight of FO or Oro Inca (OI) oil (rich in ALA). The results showed that innate immune cells enhanced phagocytic capacity and increased processing and removal of antigens. It was also observed that there was a reduction in the production of pro-inflammatory cytokines [tumor necrosis factor-alpha (TNF-α) and interleukin 6 (IL-6)] by macrophages. Lymphocytes showed a reduction in its proliferative capacity, increased differentiation of CD8^+^ subpopulation, and increased TNF-α production. Thus, oil rich in ALA showed similar immune modulation in cancer when compared with FO (77).

Kaveh et al. (78) studied the effects of ALA on sensitized rats and measured the tracheal responsiveness, total protein, phospholipase A2, IgE, IL-4, INF-γ level, and INF-γ/IL4 ratio in bronchoalveolar lavage fluid. They reported that the immune-modulatory effect of ALA increased INF-γ and INF-γ/IL4 ratio, and decreased IL-4 in sensitized rats. ALA also had a preventive effect on inflammatory markers and tracheal responsiveness.

## Studies in Ruminants

In recent times, the importance of identifying physiologically functional ingredients in ruminant milk and dairy yields for their nutraceutical and health benefits and also to meet the rising consumer demand has gained the attention of researchers worldwide. Enhancing the milk FA profile is an innovative strategy to further enhance the physiological and nutritional characteristics of ruminant milk for benefiting animals and humans. Ruminant diets are generally low in lipid content. About 60–70% of dairy milk fat consists of SFAs, 20–35% monounsaturated fatty acids (MUFAs), and only 5% PUFAs. This is mainly due to the rumen biohydrogenation process—a major portion of the PUFAs (60–90%) experience hydrolysis and isomerization reactions and lead to saturation. Studies have established that objects provided with fresh forages and grazing grass yield milk with a greater content of PUFAs in comparison with those animals fed fodder. Lipid supplements have been found to modulate the acid composition of milk effectively. Ruminant diets are poor in EPA and DHA content and hence present in very minute quantities in milk (<0.1% of total fats) (79).

Omega-3 anti-inflammatory and immunomodulatory abilities have been made use of in the study on animals to produce animal resources characterized by specific nutraceutical properties. Many past kinds of research have been done *in vitro* and *in vivo* to evaluate the immunomodulation of supplementary n-3 PUFAs in goats. To elaborate on a few, goats supplemented with FO diets during their transition period showed skin feedback to a phytohemagglutinin (PHA) skin challenge after kidding, thus showing high levels of cell-mediated immunity. Circulating lymphocytes were found to be high which also proved that FO was effective. Also, n-3 FAs modulate the CD4^+^/CD8^+^ leukocyte proportion through peripartum, supporting the CD4^+^ subset (T-helper lymphocyte cells). On the other hand, corn oil supplementation lowered the expression of CD49d (α-4 integrin)—a subsection of leukocyte homing receptors that change tissue-specific cell metabolic pathways. *In vitro* studies analyzed the function of leukocyte in fat-supplemented goats. It was observed that neutrophils taken from dairy goats generated fewer ROS when administered doses (25, 100, 200 μM) of DHA, but not of EPA. A very low dosage (25 μM) stimulated neutrophil phagocytic activity. Similarly, another study in goat monocyte-isolated cells showed that phagocytic activity was the highest for low to intermediate levels of EPA (25–100 μM) and only intermediate levels of DHA (100 μM), whereas greater levels of both EPA and DHA proved to be ineffective and also detrimental on cultured monocyte cells as showcased by expansion in caspase-7 and caspase-3 pro-apoptotic activity (79). Farina and colleagues also previously reported studies that showed dietary FO ingested by transition dairy goats was beneficial to cell-mediated immune function. DHA from FO caused an increase in PMN phagocytic activity and lowered ROS production *in vitro* and under *in vivo* conditions when the effects of EPA and DHA were studied; it showed that goat health improved by the action of neutrophils and thus prevented damage at the cellular and tissue levels by ROS. EPA and DHA also influenced goat monocytes by the upregulation of phagocytosis and ROS making by interfering with lipid droplet development and upregulating proteins of the PAT protein family (80). Caroprese et al. also took up a study to assess diets supplemented with FO and linseed oil on the immune profile of 24 Garganica grazing goats. They analyzed the *in vivo* immune responses by monitoring cell-mediated and humoral immune responses to check for the effects of PUFAs. Linseed milk showed lower SFA and higher PUFA than FO milk. Linseed and FO administration lowered humoral immunity but did not affect cellular immunity. Dietary linseed supplementation in grazing goats resulted in modulation of immune responses (81).

During the transition phase in Holstein cows, supplementation with SO induced a pro-inflammatory state indicated by the enhanced neutrophil expression of adhesion molecules, synthesis of cytokines, increased bactericidal action, and enhanced circulating acute-phase proteins. Supplementation with FO after 35 days post partum (dpp) stimulated an anti-inflammation that was demonstrated by the attenuation of neutrophil cytokine manufacturing.

SO is a fat fortifier rich in LA; it can lower the threshold for triggering an immune action that changes innate immunity. This pro-inflammation might be suitable for coping with the hectic and greatly contaminated postpartum period. Conversely, FO can enhance the threshold for triggering an immune action throughout the breeding period, exerting an anti-inflammatory status that might weaken the immune functions in early pregnancy upon environmental triggers (i.e., mastitis, heat stress) that may help the well-being of the embryo (82).

Silvestre et al. (82) studied 3,500 Holstein cows supplemented with calcium salts (CS) of palm oil (PO) or safflower (SO) from 30 days prepartum until 35 dpp and CS of PO or FO from 35 to 160 dpp. This was done as an attempt to promote immune response during the transition by using n-6 FAs compared with n-3 FAs. The percentage of neutrophils with phagocytic and oxidative burst activities was not influenced by transition diets, but activities per neutrophil were higher in SO, relative to PO diets at 4 (phagocytosis and oxidative burst) and 7 dpp (oxidative burst). This enhancement in phagocytosis of bacteria by neutrophils could be a result of the enhancement of n-6 precursors of eicosanoids or a decrease of anti-inflammatory n-3 FAs. This study also revealed an enhancement in plasma concentrations of two acute-phase proteins; this also revealed that the liver was inflamed during this period. This influence of neutrophils and acute-phase proteins was thought to enhance the immune response and in turn decrease infection; however, it is still unclear if this would be beneficial for a transition cow (83).

Didara et al. (84) studied 20 dairy Holstein cows fed isoenergetic and isonitrogenous diets; soybean meal from control (C) group was replaced with flaxseed in the experimental (FLS) group. The phagocytic activity of cows fed with n-3 FAs was significantly decreased (*P* < 0.05) relative to the group of cows provided with n-6 FAs. He reported that marked immune suppression was observed during the periparturient period in Holstein cows. Hence, dietary n-3 FAs along with oxidative stress contributed to the reduced cellular immunity throughout the periparturient period.

A total of 45 Holstein cows were fed diets that consisted of a blend of Ca salts of fish, safflower, and palm oils to make three different proportions of n-6 to n-3 FA; namely, 3.9, 4.9, or 5.9 parts of n-6 to 1 part of n-3 FA. Phagocytosis and oxidative burst by neutrophils harvested from circulation were unaffected by dietary feed ration in the first 48 h after intramammary LPS infusion. Feeding Holstein cows more n-3 and less n-6 FA in their dietary feed ration attenuated the acute-phase response after the intramammary challenge with lipopolysaccharide (LPS), although the diet did not reduce losses in intake and production during the inflammatory challenge (85).

The results obtained by Gandra et al. (33) are in line with the idea that PUFA supplementation can modulate innate and adaptive cellular immunity by increasing phagocytic capacity and activity of monocytes and by enhancing the production of adhesion molecules in T lymphocytes. In addition, an increase of expression of adhesion molecules in response to PUFA supplementation suggests a proinflammatory effect of PUFA in transition dairy cows. The n-3 FA appears to have a greater influence on phagocytic capacity and activity of leukocytes than n-6 FA.

The n-3 and n-6 FAs may be an effective tool to encourage inflammation of transition cows wherein omega-3 supplementation repressing inflammation to promote productivity and omega-6 supplements to improve immunity and reduce infectious diseases. Researchers must continue to pursue the best strategy to address challenges in farms (86). Constant research in ruminant lipid metabolism along with knowledge on the action of FAs has led to their use in modulation of animal products like enhancing the unsaturated FA content. A careful selection of lipid sources should be taken into consideration when supplementing it into the diets to enrich the acidic content of milk. At the same time, the inhibitor effect of PUFAs on ruminal metabolism has been seen in the reduction of milk fat content (MFD). This phenomenon is lesser in goats than in sheep and cows. To enhance the efficacy of PUFAs in milk, a worthy approach could be using rumen-protected or rumen-inert fat complements. Dietary fats show modulation related to metabolic, inflammatory, and immune functions, via direct action on receptors and transcription factors and via their bioactive oxygenated metabolites as well. The immunomodulatory activity of fats showing improved functionality in goats is clear. An assumption can be made for the beneficial effects of PUAs on host defense from non-specific immune stimuli during metabolic and immune stress during the peripartum phase. Molecular analyses have shown to be evasive and thus ineffective in recognizing relationships between supplementary diets and inflammatory agents and immunity-related genes (87).

In ruminants, milk FA composition can be improved through their nutrition which in turn has a positive impact on human health through the consumption of dairy products enriched in omega-3 FAs and LA. Omega-3 FAs have proven to modulate immune and inflammatory response in dairy ruminants. Feeding pregnant animals with these bioactive FAs can impact its progeny health status. Dietary long-chain PUFAs can also affect changes in lipid metabolism gene network in organs such as liver, adipose tissue, and mammary gland and temporal modulation on lipid metabolism (88).

The lactation capacity of ewes and goats constitutes ~50% of the daily energy used which generates a negative energy balance which further affects the metabolism and reduces energy which is essential for the regulation of lactation and other reproductive processes. The maximum productivity of lactating animals can be obtained by regulating energy content and resources. Ewes supplemented with PUFAs in the form of palm oil showed improvement in the number and sizes of pre-ovulatory follicles, ovulation rates, conception, and lambing rates. So a study used 30 multiparous lactating ewes (Rahmani × Barki) to examine the effects of two dietary supplemental energy sources on their metabolic attributes, milk production, and ovarian activity of ewes during early to mid-postpartum period. They were given diets supplemented with palm oil or sugarcane molasses. The results showed that both energy sources lowered body weight. Overall, this fat supplementation increases serum triglycerides which provided energy sources for enhancing milk productivity. The fatty acid profile also boosted the quality of the ovulatory follicle and the ovulation process (89). Hashem and El-Zarkouny (90) examined the effects of short-term supplementation with rumen-protected fat of 76 Rahmani, Barki, and Awassi × Barki during late luteal phase on reproduction and metabolism. They concluded that short-term supplementation with rumen-protected fat helped to improve metabolism, conception, and lambing rates of ewes during breeding.

## Studies in Humans

Dietary FAs and their by-products have an impact on human health and health outcomes. The study of dyslipidemia by lipidomic analysis showed that SFAs and n-6 PUFA are inflammatory in nature. The rise in diseases like type II diabetes, nervous system diseases, cardiovascular disorders, and atherosclerosis associated with unhealthy diets has led to the significance of lipid homeostasis in health and disease. N-3 and n-6 FAs are essential FAs as the body is not able to synthesize them. N-3 FAs are anti-inflammatory, whereas n-6 FAs are pro-inflammatory. The conversion of AA to inflammatory mediators by COX-2 gives rise to resolution and therefore EPA and DHA generate pro-resolution lipids. Also, these FAs alter signaling pathway recognition receptors and G protein–coupled receptors (GPR40) on lymphocytes, which in turn decreases inflammatory risks in the case of cardiovascular disorders. LC-PUFA metabolites, otherwise known as eicosanoids, interact with GPCRs and have been related to the development of atherosclerosis. Intake of dietary omega-3 FAs offers precursors for the synthesis of anti-inflammatory lipids called leukotrienes such as LTB_5_ and prostanoids PGH_3_ whereas a rise in n-6 PUFAs produces LTA_4_, LTB_4_, LTC_4_, LTD_4_, LTE_44_, and PGH_2_. The primary inflammatory response in the human body, characterized by the synthesis of LTB_4_, is essential for neutrophil infiltration to the infection site and then proceeds with a cascade of cytokine production. These bioactive derivatives are incorporated into the membrane phospholipids and influence membrane fluidity and surface receptor expression. There are some studies on the inclusion of FAs into the cell membrane of macrophages as seen in activated polymorphonuclear neutrophils (PMNs) where there is a loss of PUFAs as a result of the very high intracellular phospholipase activity (cPLA2) due to leukocyte stimulation (41).

Since the 1980's, it has been well-known that preventing the lipoxygenase metabolism of inflammation inhibits the differentiation of monocytes to macrophages after administration of AA. Fortification of cells with n-3 FAs like linolenate (FA 18:3 n-3) stimulates a faster immunity by decreasing TNF-alpha in contrast to macrophages fortified with n-6 FAs like LA, depending on macrophage development. Omega-3 FAs also help in expressing the Mac-1 complex (CD-11b/CD-18) on the neutrophil membrane. The classically activated pro-inflammatory M1 macrophages are rich in LTB4 and PGE2 whereas M2 macrophages are rich in pro-resolving mediators from the 5-lipoxygenase pathway (5-LOX) and eicosanoids from n-3 FAs like EPA and DHA such as resolvin D2 and D5 (RvD2, 5). M2 cells produce higher levels of PGD2, the pro-inflammatory lipid mediator downstream of the AA/COX pathway (41).

Some *in vitro* studies in humans showed decreased phagocytosis after exposure to n-3 PUFAs. Sipka et al. (91) cultured neutrophils of healthy subjects with zymosan and 20 μg/ml of EPA. The results showed that EPA decreased chemiluminescence (emission of photons as a result of phagocytosis) by about 50%. In the same study, the authors investigated plasma membrane fluidity by labeling of neutrophils with propionic acid during EPA addition. In non-stimulated neutrophils, EPA enhanced the flexibility of cell membranes. Conversely, in zymosan-activated cells, there was a small period of fluidization after which EPA resulted in a significant rigidification of the plasma membrane. This rigidification was explained by the authors to be an explanation for the reduced phagocytic activity and chemotaxis of human neutrophils.

Versleijen et al. (92) cultured healthy human neutrophils with opsonized *Streptococcus pneumoniae* and 5 mmol/L emulsions derived from soybean (LCT), fish (VLCT), olive (LCT-MUFA), a blend of soybean/coconut oils (LCT/MCTs), or structured lipids (SL) for 1 h. The authors observed decreased phagocytosis after LCT-MUFA (70 ± 6%), VLCT (67 ± 2%), SL (63 ± 9%), LCT (66 ± 10%), and LCT/MCT (47 ± 15%) relative to the no lipid control (75 ± 3%).

Dietary intake in humans has been suggested to alter the human ability to synthesize LC-PUFA essential C18 FA precursors, i.e., ALA and LA. However, these studies in general show little evidence of an influence of n-3 PUFA on phagocytic activity. Seven studies showed no effect (93–99), two studies showed a detrimental effect (100, 101), and one study showed enhancement of phagocytosis after feeding n-3 PUFA (102).

In the study of Schilling et al. (97), the authors fortified patients with major abdominal surgery with an enteral formula rich in n-3 PUFA for 4 days and reported no effect on phagocytosis of *E. coli* by PBMCs. Halvorsen et al. (98) revealed no influence of EPA or DHA per day for 7 weeks on the phagocytic activity of opsonized or un-opsonized bacteria by monocytes, although there was a trend toward greater attachment of monocytes to bacteria after feeding EPA. Similarly, Thies et al. (96) supplemented healthy subjects with nine capsules of placebo oil (an 80:20 blend of palm and sunflower oils) or mixes of placebo oil with oils rich in ALA (2 g) or γ-linolenic acid (770 mg) or arachidonic acid (680 mg) or DHA (720 mg) or a mix of EPA plus DHA (720 mg of EPA + 280 mg of DHA), daily for 12 weeks. Results of the phagocytosis test showed no effect of supplementation on phagocytosis of PMBCs enhanced with opsonized FITC-labeled *E. coli*. Moreover, 4.5 or 9.5 g α-linolenic acid, and 0.77 or 1.7 g EPA+DHA, daily for 6 months did not affect phagocytosis of healthy PBMCs to opsonized FITC-labeled *E. coli* (94). Kew et al. (99) supplemented healthy humans with 4.75 g EPA/day + 0.73 g DHA/day, or 0.85 g EPA/day + 4.91 g DHA/day for 4 weeks and reported no effect on the phagocytic activity of PBMCs to opsonized FITC-labeled *E. coli*. In the study of Miles et al. (95), there was no influence of supplementing healthy subjects with EPA and stearidonic acid on their PBMC phagocytosis capacity. Rees et al. (93) investigated the influence of increasing concentrations of EPA (1.35, 2.7, or 4.05 g/day) for 12 weeks on phagocytic activity in young and old healthy objects and showed no effect of EPA.

Exceptionally, two studies showed decreased phagocytosis (100, 101). In the study of Virella et al. (100), the authors supplemented fit humans with 6 g/day of a FO extract (equivalent to 2.1 g of EPA/day) per day for 6 weeks and showed reduced phagocytosis of PBMCs to *E. coli*. Margaro et al. (101) supplemented rheumatoid arthritis patients with 1.6 g EPA and 1.1 g DHA/day for 45 days and observed reduced chemiluminescence of neutrophils stimulated by zymosan (6.02 ± 2.89 vs. 7.7 ± 2.69) and by PMA at 21 days (5.19 ± 2.5 vs. 7.01 ± 3.31) and 45 days (5.08 ± 1.93 vs. 7.01 ± 3.31), compared with basal values.

To date, evidence in humans has shown that anti-inflammatory effects of n-3 PUFAs were first recognized in epidemiological studies in Eskimos as they consumed a lot of fish which is rich in n-3 LC-PUFAs. Some clinical studies showed the positive influence of n-3 LC-PUFAs in chronic inflammatory and autoimmune diseases. FO decreased pro-inflammatory cytokine expression such as IL-1β in blood monocytes and enhances the symptoms of patients suffering from rheumatoid arthritis or multiple sclerosis. DHA reduced circulatory inflammatory markers and oxidative stress. It also helped patients with moderate cognitive alterations (103).

The appearance of atherosclerotic lesions as a trigger due to an increase in concentrations of cholesterol-rich lipoproteins is distinguished by inflammatory conditions. Of late, it was seen that blocking pro-inflammatory metabolic pathways without altering lipid concentrations can decrease the danger of heart disorders. A diminished vascular inflammatory response is a critical factor in atherosclerosis, and the production of pro-resolving lipid mediators is considered as a defensive mechanism against murine atherosclerosis. EPA metabolic pathways result in the formation of 8-monohydroxy EPA (18-HEPE), the precursor for the lipid mediator resolvin E1 (RvE1), which transduces its resolving actions using the G protein–coupled receptor named as ERV1/ChemR23. Hence, an investigation was started to further explore the metabolic pathways associated with EPA fortification and synthesis of lipid mediators that mediate atherosclerotic disease progression. Studies in the literature revealed that fortification with EPA significantly weakened atherosclerotic lesion growth caused by western diet in these mice and was linked with cardiovascular n-3 PUFA supplementation and charged lipoprotein (104).

The anti-inflammatory properties of n-3 PUFA are a result of their ability to inhibit the production of inflammatory mediators such as eicosanoids (PGE_2_, 4-series leukotrienes), pro-inflammatory cytokines (IL-1β, TNF-α, IL-6), chemokines (IL-8, MCP-1), adhesion molecules (ICAM-1, VCAM-1, selectins), platelet-activating factor, and reactive oxygen and nitrogen species. Besides inhibition of pro-inflammatory mediators, n-3 PUFA can increase the production of an anti-inflammatory cytokine such as IL-10. The anti-inflammatory action of n-3 PUFA is governed by modulation of gene activation. This gene activation is regulated by nuclear factor-kappa B (NF-κB), a transcription factor ubiquitous in almost all cell types. One of the striking characteristics concerning n-3 research is the discovery of pro-resolution agents by serving as the precursors for several families of pre-resolving mediators, which at least include EPA-derived E-series resolvins, DHA-derived D-series resolvins, and DHA-derived protectins and maresins. Limited data are surrounding human immunomodulatory and anti-inflammatory actions of resolvins and maresins (105).

## Concluding Remarks and Future Perspectives

Dietary PUFAs play a role in innate and adaptive immunity as described in *in vitro* studies and animal models. However, from a clinical point of view, the results obtained are uncertain as many diseases in humans are dependent on genetic and environmental factors. In addition, experimental techniques to analyze the effects of FAs on immune responses lack clarity and give varying results. The doses utilized in *in vitro* and *in vivo* studies are not in line with physiological concentrations in biological fluids and tissues (106).

PUFAs have been a topic of interest since the late 1980's. This area of research falls into different categories, predominantly in humans. Although the information in this area is extensive, there are significant gaps and inconsistencies in studies to be able to come to a firm conclusion. A reason for this could be the variation in responses in a population or other factors like dietary components, sex, experimental species, age, body composition, and genetics. These factors have been currently researched with advancements in analytical and statistical technologies. Focus is mostly put on EPA and DHA studied in combination and not as two separate entities. It is well-known by now that fish and FO are rich sources of n-3 PUFA. However, the supply of fish for human consumption is not sustainable and thus alternate sources are being researched such as algal oils and seed oils (107). N-6 PUFA is less significant and is mostly used as the control for n-3 PUFA in studies.

ALA, DHA, and EPA mostly exert an inhibitory effect on the activation of immune cells from innate and adaptive immunity. Some specialized immune functions are promoted by dietary omega-3 FAs in certain immune cells, such as phagocytosis by macrophages and neutrophils or Treg differentiation which indicates that omega-3 FAs do not act as unspecific immune repressors. Varying data from numerous studies give rise to different approaches for the incorporation of FAs in *in vitro* cellular cultures, for example, differences in enrichment doses or incubating time, length of fortification time, etc. Reports on mouse models may vary with that in humans as both species are different immunologically and at the metabolic level. Fortification of n-3 FAs in human diets have lesser concentrations of EPA and DHA and are present for a shorter duration period than in mouse experiments. Also, mouse diets are controlled by the researchers whereas the human diet is complicated and varied (1).

Based on the aforementioned, dietary PUFAs may or may not modulate the immune system by affecting phagocytosis. Care should be taken when these FAs are supplemented to ensure the general health. The effect of FAs on murines either showed a lowering effect on phagocytosis or had no effect. In ruminants, the n-3 FAs seem to have a significant influence on phagocytosis. Overall, in humans, most studies showed no effect except a few, which points in the direction of more in-depth research.

## Author Contributions

HA-K wrote the manuscript and the author confirms being the sole contributor of this work and has approved it for publication.

## Conflict of Interest

The author declares that the research was conducted in the absence of any commercial or financial relationships that could be construed as a potential conflict of interest.
